# Benzothiazinethione is a potent preclinical candidate for the treatment of drug-resistant tuberculosis

**DOI:** 10.1038/srep29717

**Published:** 2016-07-13

**Authors:** Chao Gao, Cuiting Peng, Yaojie Shi, Xinyu You, Kai Ran, Lu Xiong, Ting-hong Ye, Lidan Zhang, Ningyu Wang, Yongxia Zhu, Kun Liu, Weiqiong Zuo, Luoting Yu, Yuquan Wei

**Affiliations:** 1 State Key Laboratory of Biotherapy/Collaborative Innovation Center for Biotherapy, West China Hospital, West China Medical School, Sichuan University, Chengdu, 610041, Sichuan, China; 2 Department of Pharmaceutical and Bioengineering, School of Chemical Engineering, Sichuan University, Chengdu, 610041, Sichuan, China; 3 Department of Respiratory and Critical Care Medicine, West China Hospital, Sichuan University, Chengdu, 610041, Sichuan, China

## Abstract

New chemotherapeutic compounds are needed to combat multidrug-resistant *Mycobacterium tuberculosis* (*Mtb*), which remains a serious public-health challenge. Decaprenylphosphoryl-β-D-ribose 2′-epimerase (DprE1 enzyme) has been characterized as an attractive therapeutic target to address this urgent demand. Herein, we have identified a new class of DprE1 inhibitors benzothiazinethiones as antitubercular agents. Benzothiazinethione analogue SKLB-TB1001 exhibited excellent activity against *Mtb* in the Microplate Alamar blue assay and intracellular model, meanwhile SKLB-TB1001 was also highly potent against multi-drug resistant extensively and drug resistant clinical isolates. Importantly, no antagonism interaction was found with any two-drug combinations tested in the present study and the combination of SKLB-TB1001 with rifampicin (RMP) was proved to be synergistic. Furthermore, benzothiazinethione showed superb *in vivo* antitubercular efficacy in an acute *Mtb* infection mouse model, significantly better than that of BTZ043. These data combined with the bioavailability and safety profiles of benzothiazinethione indicates SKLB-TB1001 is a promising preclinical candidate for the treatment of drug-resistant tuberculosis.

Tuberculosis (TB) continues to be a major health threat affecting the world today. According to the World Health Organization (WHO) report^1^, there were 9.6 million active TB cases and 1.5 million TB-induced deaths in 2014. The current drug therapy regimen for TB was proposed 40 years ago^2^. However, long duration of therapy, severe side-effects and issues of non-compliance have hindered this regimen and the appearance of drug resistance has worsened this situation^3^,^4^,^5^.

In response to the emergence of multi-drug resistant (MDR) and extensively drug resistant (XDR) strains of *Mycobacterium tuberculosis* (*Mtb*), numerous initiatives and efforts are being undertaken to combat this global killer. There appears some new therapeutic anti-tuberculosis agents especially in the past 5–6 years after several decades of near inactivity to fill the drug pipeline^6^,^7^,^8^,^9^,^10^. And recently, Sirturo (bedaquiline, also known as TMC207) and delamanid (OPC67683) were approved as part of combination treatment for MDR tuberculosis^11^,^12^,^13^. While there will be a time for vastly expanded access to these new agents due to some safety risks, and the search for effective clinical candidates is vital in order to sustain the tuberculosis drug pipeline^14^,^15^,^16^.

Decaprenylphosphoryl-β-D-ribose 2′-epimerase (DprE1) have been characterized as one of the most attractive targets for anti-tuberculosis drug discovery^17^,^18^,^19^. As the components of decaprenylphosphoryl-β-D-ribose 2′-epimerase complex, DprE1 and DprE2 catalyze the epimerization of decaprenylphosphoryl-β-D-ribose (DPR) to decaprenylphosphoryl-β-D-arabinofuranose (DPA) which is an important precursor for the synthesis of mycobacterial cell wall arabinans^20^. Some new chemical entities (NCE) are proven to be potent against MDR/XDR-TB as covalent or non-covalent inhibitors of the DprE1 enzyme^21^,^22^,^23^,^24^,^25^,^26^. *Nitro*-benzothiazinones (BTZs) are known the most advanced scaffold among these NCEs. And BTZ043, which acts by forming a covalent bond with Cys387 in the active site pocket of DprE1, is nearing phase I clinical trials with a MIC of 1 ng/mL^20^. More recently, the pre-clinical candidate arose from lead optimizations, PBTZ169, has shown great promise for the treatment of tuberculosis and is poised to enter clinical trials^27^.

The SAR studies and the detailed mechanistic studies indicate that the NO_2_ group at position 8 and the sulfur atom at position 1 were critical for activity and the trifluoromethyl at position 6 also plays an important role in maintaining anti-tubercular activity^20^,^28^,^29^,^30^. In one of our endeavors to find a new antitubercular agent, we discovered a new series of benzothiazinones with satisfactory antimycobacterial properties^31^,^32^. Inspired by the promising results from BTZ043, PBTZ169 and the antitubercular potential of the sulfur rich heterocycles in our earlier studies, we herein explored the antitubercular potential of a new sulfureous scaffold benzothiazinethione.

## Results

### Identification of benzothiazinethiones

**Figure 1:**
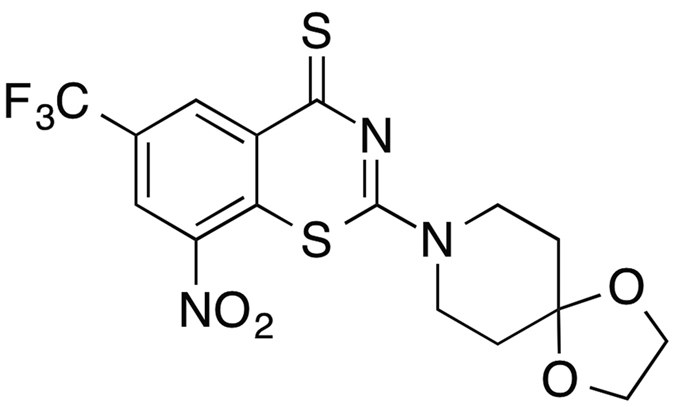
The structure of compound SKLB-TB1001.

Benzothiazinethiones were obtained from the relevant benzothiazineones (BTZs), and the general synthetic route of BTZs can be found in previous studies^31^,^32^. We investigated a series of compounds with representative properties and used the microplate alamar blue assay to screen for activity against *M. tuberculosis* (Supplementary Table S1). One compound in particular, 8-nitro-2-(1,4-dioxa-8-azaspiro[4.5]decan-8-yl)-6-(trifluoromethyl)-4H-benzo[e][1,3]thiazine-4-thione (SKLB-TB1001) (Fig. 1), was found to be equipotent to BTZ043 and PBTZ169 against replicating H37Rv (Table 1) and more potent than isoniazid (INH). These compounds were evaluated for cytotoxicity for Vero, A549 and J774A.1 cells. SKLB-1001 had relatively high 50% inhibitory concentrations (IC_50_s), indicating relatively good selectivity. When considering such factors as manufacturing, physicochemical and pharmacokinetic properties along with the antimicrobial activity *in vitro* (Supplementary Tables S1 and S2), SKLB-1001 was selected for further study.

The anti-tubercular activity was also tested on 5 clinically isolated strains (Table 2). Notably, SKLB-TB1001 was similarly active against MDR clinical isolates of *M.tb* showing resistance to isoniazid and rifampicin in addition to streptomycin and also equivalently potent for XDR strains which were resistant to all tested drugs. The results of our evaluation indicated that SKLB-TB1001 exhibits satisfactory anti-mycobacterial activity on both drug-susceptible and drug-resistant strains, suggesting it has no cross-resistance with any of the currently used anti-TB drugs and its potential for use against drug-resistant *Mtb* strains.

As shown in Table 1, *M. avium* was resistant to SKLB-TB1001 and BTZ043, consistent with previous studies on DprE1 inhibitors. The computational docking and differential scanning calorimetry (DSC) of DprE1 with SKLB-TB1001 also indicated the inhibition of DprE1 by SKLB-TB1001 (Supplementary Figs S1 and S2).

**Figure 2:**
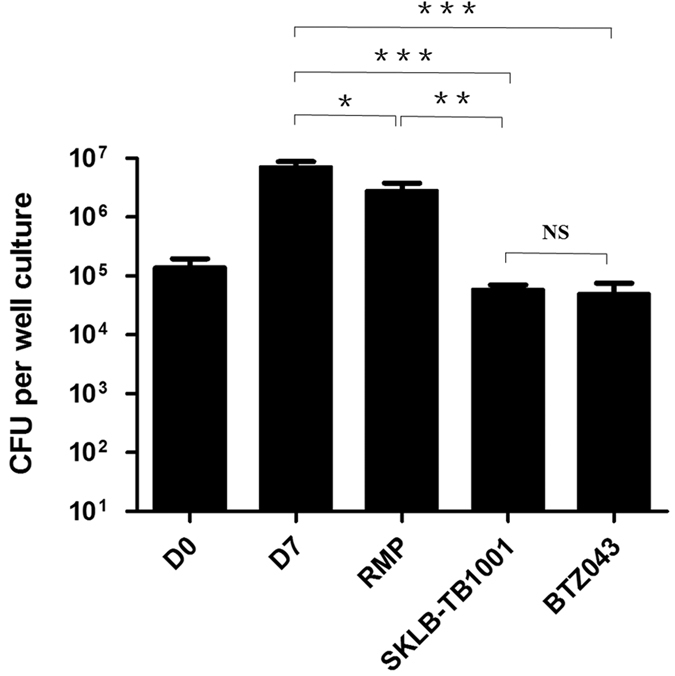
Antimicrobial activity of SKLB-TB1001 against *M. tuberculosis* Erdman in J774A.1 cells. Triplicate cultures were treated with antitubercular agents at 0.02 μg/mL. Values represented mean ± SD. D0 and D7 represent the CFU counts of untreated control at the beginning and end of incubation, respectively. *P* values for comparison of two groups were determined by 2-tailed Student’s *t* test (*P < 0.05; **P < 0.01; ***P < 0.001 vs D7 group; NS means having no statistically significant).

In addition, SKLB-TB1001 inhibits growth of intracellular *Mtb* (Fig. 2). The bactericidal activity against *M. tuberculosis* Erdman in J774A.1 macrophages was observed for SKLB-TB1001 as well as for BTZ043 and rifampin controls. SKLB-1001 and BTZ043 effected a 2.1 and 2.2 log reduction in viability at 0.02 μM, respectively, while rifampin demonstrated more modest activity (Supplementary Table S3).

### Interaction of SKLB-TB1001 with rifampin, isoniazid, moxifloxacin and linezolid

The interaction of SKLB-1001 with chemically diverse antitubercular agents was examined *in vitro* using checkerboard synergy assay (Table 3). The interaction between two agents in combination can be described as synergistic, additive and antagonistic. Table 3 lists the MIC of each individual drug, as well as fractional inhibitory concentration index (FICI) of SKLB1001 in combination with front-line and experimental anti-TB agents. The data indicate that the combination of SKLB-TB1001 and RMP was synergistic (FICI = 0.22): MIC of SKLB-TB1001 in the presence of RMP was decreased 5-fold, and the MIC of RMF in the presence of SKLB-TB1001 was decreased 6-fold. Nevertheless, FICI of BTZ043 in combination with RMP was 0.85 suggesting an additive interaction. The MIC of SKLB-1001 in the presence of isoniazid or moxifloxacin was clearly lower than the MIC when it was used alone (FICI = 0.68, 0.97, respectively). The combination of SKLB-TB1001 and linezolid caused a FICI of 1.34 indicating that the drugs act additively against *M.tb*. No antagonism interaction was found with any two-drug combination tested in the present study.

### Pharmacokinetics of SKLB-TB1001

**Figure 3:**
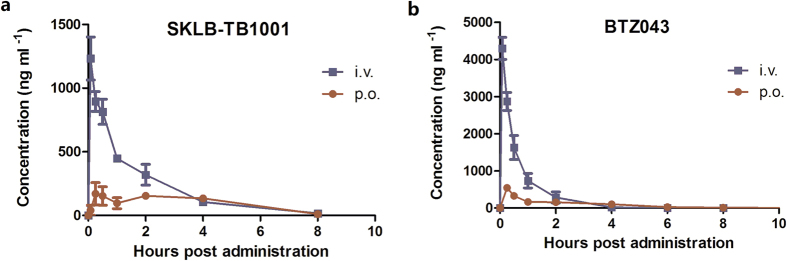
Pharmacokinetic analysis of SKLB-TB1001 in SD rats. Mean plasma concentration-time curves of (**a**) SKLB-TB1001 and (**b**) BTZ043 following a single 5 mg/kg dose administered by oral gavage (p.o.) or intravenous injection (i.v.) in SD rats. Data are presented as means ± SD (n = 3).

Pharmacokinetic properties of benzothiazinethione analogue SKLB-TB1001 and benzothiazineone analogue BTZ043 were investigated in SD rats (Table 4, Fig. 3). Oral administration of SKLB-TB1001 and BTZ043 at 5 mg/kg resulted in rapid absorption (T_max_ 1.13 and 0.25 hours) and moderate elimination half-life (t_1/2_ 1.45 and 1.22 hours). The comparison of peroral AUC with intravenous AUC of SKLB-TB1001 in plasma over time resulted in an apparent oral bioavailability of 44.4%, which was significantly higher than that of BTZ043 (F 29.5%). These results indicated that benzothiazinethione analogue SKLB-TB1001 have adequate PK properties enabled *in vivo* efficacy.

### Efficacy of SKLB-TB1001 in the acute *Mtb* Erdman infection mouse model

**Figure 4:**
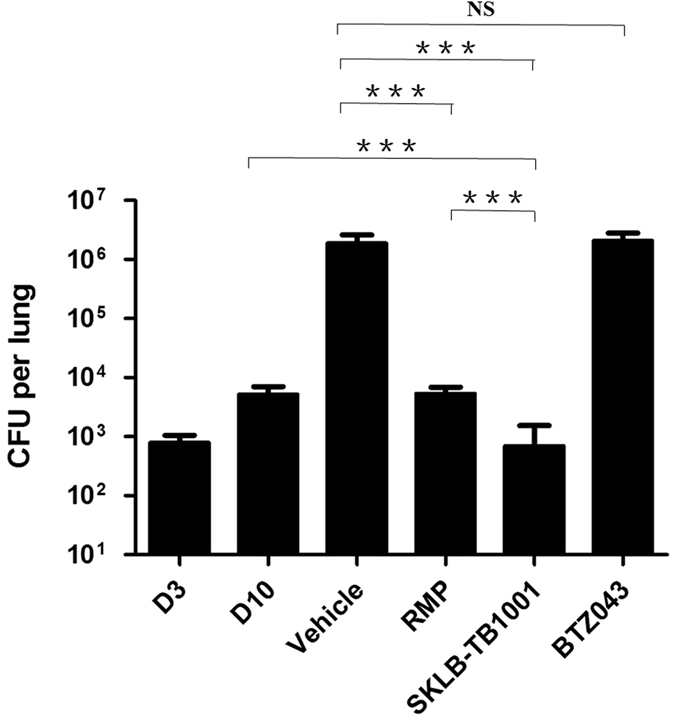
Efficacy of SKLB-TB1001 in *Mtb* Erdman infected mice compared with RMP. The bars indicate mean ± s.d. CFU counts in each group (n = 6 for treated groups and n = 5 for untreated control). The columns, D3 and D10 shows bacterial loads in control mice 3 days and 10 days after infection, respectively. *P* values for comparison of two groups were determined by 2-tailed Student’s *t* test (*P < 0.05; **P < 0.01; ***P < 0.001; NS means having no statistically significant that is *P* > 0.05).

Table 5 and Fig. 4 showed the activity of antitubercular drugs on mice infected with *M.tb* Erdman. Oral RMP at 15 mg/kg was positive control. RMP reduced bacterial concentration in lungs 2.5 logs, compared to bacterial concentration in tissues of vehicle control group. Encouragingly, SKLB-TB1001 treatment of infected mice at 150 mg/kg daily for 4 weeks resulted in a reduction of *M.tb* CFU in lungs by 3.4 logs compared to the vehicle group and 0.87 logs reduction compared to treatment initiation, which was clearly more effective than RMP at 15 mg/kg(*P* < 0.001). In mice treated with 150 mg/kg of SKLB-TB1001, the *M.tb* burden was reduced by the treatment to the same level of CFU as in the untreated controls at day 3 after infection. Particularly, BTZ043 was not significantly active at 150 mg/kg in this model.

### Acute toxicity study

The maximum non-toxic dose of SKLB-TB1001 *in vivo* is greater than 1 g/kg in BALB/c mice. There was no death in acute single dose toxicity studies in BALB/c mice. All the mice behaved normally when administering by celiac injection. No significant changes in general appearance or behavioral pattern were noted till the end of 14 days. No statistically significant differences in body weight, viscera, biochemical parameters and Hematology index between treatment group and control group (Table 6, Supplementary Fig. S3). No observable pathological changes were found in heart, liver, lung, kidneys and spleen (Supplementary Fig. S4).

## Discussion

New agents and therapeutic methods for TB, especially MDR- and XDR-TB, are greatly needed in the global effort to control this deadly disease^33^. DprE1 enzyme and Benzothiazinone analogues have displayed some promising results for tuberculosis treatments. We described herein a new scaffold, benzothiazinethione, which was obtained from the thionation of benzothiazinone and provided new insight into the structural and pharmacological requirements for DprE1 inhibitors as potent antitubercular agents. Generally, benzothiazinethiones maintained the antitubercular activity at different levels. In the series of studies described here, SKLB-TB1001, which was equivalent to BTZ043, exhibited potent activity against either *M. tuberculosis* standard strain or drug-resistant strains *in vitro*. Despite its low aqueous solubility, SKLB-TB1001 exhibited favorable PK properties after oral administration. In the acute infection mouse model, after 4 weeks of drug treatment, the efficacy of SKLB-TB1001 was more obvious than that for rifampicin and was better than that for BTZ043. In the *in vitro* combination study, no antagonism occurred and synergistic or enhanced interactions predominated. These results demonstrated that SKLB-TB1001 is a potential preclinical candidate.

Since multi-drug therapy is essential to cure TB infections^34^, we explored the interactions between SKLB-TB1001 with the frontline TB drugs and new anti-TB drug candidates in clinical trials. No antagonism was observed between SKLB-1001 and the tested compounds, and the additive interaction was observed when SKLB-TB1001 combination with isoniazid, moxifloxacin or linezolid. Interestingly, SKLB-TB1001 showed synergy with rifampicin, while this synergistic interaction did not occur between rifampin and BTZ043 or PBTZ169 in this study and the previous reports^27^,^35^. A definitive explanation for the synergy interaction between SKLB-1001 and rifampicin against *Mtb* H37Rv is not yet available. The synergistic action in a drug collaboration depends on the drug targets and the mode of actions of the individual compounds. Perhaps the synergy was due to weaken of the cell wall by DprE1 inhibition leading to better penetration of RMP. However, the previous DprE1 inhibitors, BTZ043 and PTBZ169, did not show any synergistic interaction when used in combination with RMP in the previous studies^27^,^35^, suggesting that the effect on the cell wall structure of *Mtb* by SKLB-TB1001 does not necessarily contribute to the synergistic interaction in RMP + SKLB-1001 combination.

Hypothetically, the introduction of S atom in the BTZ structure could cause some unknown mechanism triggered by SKLB-TB1001. On the basis of the structure of benzothiazinethione and benzothiazineone, SKLB-1001 may generate some sulfur-containing metabolites or as an organic SO_2_ donor^36^,^37^. Recently, sulfur dioxide has been shown to have antimycobacterial activity and the Mtb inhibitory activity in part depended on its ability to induce stress by affecting cellular redox equilibrium and causing damage to DNA^38^,^39^,^40^. In addition, these sulfur-containing metabolites may bond to DNA or RNA due to the nucleophilicity of S atom^39^. While, these transformations occur in bacterial only or these intermediates are well tolerated in normal cells according to the high IC50s of SKLB-1001 for Vero and J774A.1 cells. Since rifampicin is a RNA polymerase inhibitor^41^, the damages to biomacromolecules caused by SKLB-1001 could exert a noticeable effect on activity of RMP. Notably, two anti-tb drugs moxifloxacin and linezolid^42^,^43^, which act on the intracellular targets of *Mtb*, did not show synergistic interaction when used in combination with SKLB-TB1001. It indicated that benzothiazinethione enhance the activity of other antibiotics selectively. Future work will include identifying the metabolites of SKLB-1001 and possible targets to fully reveal the mechanisms of benzothiazinethiones. With the *in vitro* positive (synergistic activity) drug interactions, we have initiated a series of studies with murine models of TB to confirm whether synergy also occurs *in vivo*.

In addition to its promising *in vitro* bactericidal activity, the benzothiazinethione analogue SKLB-TB1001 significantly reduced the lung CFU counts of mice after 4 weeks of treatment. Interestingly, there was no significant change in CFU counts of mice receiving BTZ043 treatment compared with control in this model. The performance of BTZ043 in the *Mtb* Erdman infection mouse model was apparently different with the previous studies which conducted in the H37Rv infection model. In the *in vitro* studies, SKLB-TB1001 and BTZ043 exhibited similar antimicrobial activity including in the *tuberculosis* Erdman infection macrophage model. Since there was a general correlation between activities in the *in vitro* and mouse models^44^, we speculated that the invalidation of BTZ043 was due to some unknown changes of pharmacological properties *in vivo*. Besides, the unique mechanism of SKLB-TB1001 may benefit enhancing its efficacy in the mouse model. In order to exclude the influence caused by *M. tuberculosis* strains used for *in vivo* infections, we are retesting the efficacy of SKLB-TB1001 against a second *M. tuberculosis* strain in mouse models. Meanwhile, work focusing on the possible mechanisms of discrepancies between BTZ043 performed in different models and the pharmacological properties of SKLB-TB1001 are in progress.

Some appreciable limitations, such as the issues of pharmacokinetics, bioavailability, safety and *in vivo* availability could result in development failures^45^,^46^. In this case, the oral bioavailability for SKLB-TB1001 in a lipophilic formulation was favorable (44.4%) and significantly higher than that of BTZ043 (29.5%), which suggested its great potential in clinical trails at higher doses. With demonstrated *in vitro* activity, SKLB-TB1001 also showed superb *in vivo* antitubercular efficacy by reducing bacterial load of 3.4 log units in lungs over a period of 4 weeks’ treatment, superior over that of RMP (15 mg/kg). In addition, *in vitro* and *in vivo* safety assessment of SKLB-TB1001 in mouse revealed a reliable security of SKLB-TB1001. Importantly, the new chemotype, benzothiazinethione, seems able to overcoming these major hurdles in preclinical drug development to some extent. When compared with benzothiazineones, it is likely that SKLB-TB1001 reflects the differences on pharmacokinetic and pharmacology properties. With these unique features of the benzothiazinethione analogue SKLB-TB1001 could be a valuable preclinical candidate.

## Methods

### Materials

The following mycobacterial strains used in assays *in vitro* were maintained in Shanghai pulmonary hospital: *Mycobacterium avium;* drug-resistant clinical isolates. *Mycobacterium tuberculosis* Erdman and *Mycobacterium H37Rv* were maintained in University of Illinois at Chicago.

Benzothiazinethiones were synthesized using previous reported methods^31^,^32^ and determined by ^1^H-NMR, ^13^C-NMR and ESI-MS analysis.

### Minimum inhibitory concentrations (MICs)

The MICs against *Mycobacterium tuberculosis* H37Rv and Erdman were determined by the microplate Alamar blue assay^47^. Compound stock solutions were prepared in Dimethyl sulfoxide (DMSO). The final test concentrations ranged from 32 to 0.0078 μg/mL. M. tuberculosis was grown to late log phase in Middlebrook 7H9 medium supplemented with 0.05% Tween 80, 0.2% (vol/vol) glycerol, and 10% (vol/vol) oleic acid-albumin-dextrose-catalase. Cultures were then centrifuged, washed, and resuspended in phosphate-buffered saline. Suspensions were then passed through an 8-um-pore-size filter to remove clumps, and aliquots were frozen at −80 °C. The number of CFU was determined by plating on 7H11 agar plates.

Two-fold dilutions of compounds were prepared in Middlebrook 7H12 medium in a volume of 100 μL in 96-well microplates. *M.tb* (100 μL inoculums of 10^6^ cfu/mL) was added, the plates were incubated at 37 °C. On the 7th day, add 20 μL 0.01% Alamar Blue and 12.5 μL of 20% Tween 80 to each well. After 24 hours the fluorescence of each well is measured at Excitation 530 nm; Emission 590 nm.

The MICs against *Mycobacterium tuberculosis* clinical isolates, *Mycobacterium avium* were determined by a microdilution plate assay^48^. Final drug concentration ranges were as follows: for *Mycobacterium avium*, 0.00125 to 64 μg/mL, for MDR-TB and XDR-TB strains, 0.0156 to 64 μg/mL. Isoniazid (INH) or RMP was used as positive control in each experiment, control wells were prepared with bacterial suspension only.

The MICs were defined as the lowest concentration effecting a reduction in fluorescence of ≥90% relative to the controls. Reported MICs are an average of two or three individual measurements.

### Checkerboard synergy assay

Briefly, in a 96-well micrometer plate, two-fold serial dilutions of compound A were added vertically. Compound B (benzothiazinethiones or benzothiazineones) was diluted horizontally, and the row and column in this plate contained individual drugs can be used calculated individual MIC. To each well of plate, a log-phase culture of bacteria was added. The plates incubated for 14 days at 37 °C prior to reading MICs. Fractional inhibitory concentrations (FIC) were calculated using the following formula: MIC in combination/MIC alone. The fractional inhibitory concentration index FICI was calculated as the sum of FIC of compound A + FIC of compound B. As outlined in previous publications about the assay, FICI scores were explained as follows: synergy (≤0.5), additivity (>0.5–4.0), or antagonism (>4.0).

### Macrophage assay

As described previously^49^, J774A.1 cells were grown in 75-cm2 cell culture flasks in 10% FBS in Dulbecco’s modified Eagle’s medium (DMEM) until confluency. The cells were detached using a cell scraper, and then centrifuged at 200 *g* for 5 min at room temperature. The cells were incubated at 37 °C under 5% CO_2_ for 16 h after distributing into 24-well plates containing coverslips. *M. tuberculosis* Erdman (ATCC 35801) were diluted to a final concentration of 1 × 10^5^ to 3 × 10^5^ CFU/mL with DMEM after thawing and sonicating from frozen cultures. And 500 ul of the dilution was dispensed to each well of a new 24-well plate. Followed by, J774A.1 cells on coverslips were infected by incubation with *M. tuberculosis* Erdman at 37 °C for 2 h. Coverslips were then washed with HBSS and transferred to new 24-well plates. The Cultures were treated in triplicate with compounds at 0.02 μM containing DMEM and incubated at 37 °C under 5% CO_2_ for 16 h. At D 0 (for untreated controls) and after 7 days of incubation, macrophage cells were lysed by addition of 200 μL of 0.25% sodium dodecyl sulfate. The plates were incubated at 37 °C for 10 min, and 200 μL of DMEM was added. The lysate was sampled using a microtube and sonicated for 15 s. Then 1:1, 1:10, 1:100, and 1:1,000 dilutions were plated on 7H11 agar plates and CFU counts determined after 2 to 3 weeks of incubation at 37 °C.

### Cytotoxicity

The compounds were examined for toxicity (IC_50_) using MTT assay^50^. Briefly, cells (3 ~ 5 × 10^3^ cells/well) were seeded in a 96-well plate. After 24 h incubation, the cells were treated with various concentrations of drugs for 24, 48, 72 h, respectively. Columns one and two in the assay plates contained-media +0.1% DMSO for a negative control ,Then, 20 μl of a 5 mg/mL MTT solution was added to each well, and the plates were incubated for an additional 2~4 h at 37 °C. The medium was subsequently discarded, and DMSO 150 μL was added to dissolve the formazan. Oscillate 15 min, the OD 570 was measured using a Spectra MAX M5 microplate spectrophotometer (Molecular Devices, CA, USA), and the IC_50_ values were calculated.

### *In vivo* acute *M.tb* Erdman infection assay

The studies were conducted by University of Illinois at Chicago and approved by the Institutional Animal Care and Treatment Committee and were carried out in accordance with the approved guidelines. Eight-week-old female BALB/c mice were infected with a suspension of approximately 6 × 10^6^ CFU of *M. tuberculosis* Erdman/mL using a Glas-Col inhalation system. Following infection, the mice were randomly divided into 6 groups and each group was composed of 5 to 6 mice. 4 mice were sacrificed to determine the CFU counts in the lungs 3 days after infection. Therapy was given daily from day 10 until day 36 postinfection. BTZ043 and SKLB-TB1001 were suspended in 0.5% carboxymethyl cellulose (CMC), and RIF were suspended in water in the study. All the drugs were administered by oral gavage in a maximum volume of 200 μL (RMP, 15 mg/kg; BTZ043 and SKLB-TB1001 150 mg/kg, respectively). The mice were sacrificed the day after the last day of treatment. Lungs were aseptically removed and were ground in a tissue homogenizer. The number of viable organisms was determined by titration on 7H11 agar plates in duplicate. The plates were incubated at 37 °C for 3 weeks before CFU were enumerated and CFU counts were log transformed before analysis.

### Pharmacokinetic study

All animal experiments have been approved by the Institutional Animal Care and Treatment Committee of Sichuan University in China and were carried out in accordance with the approved guidelines. SPF female SD rats weighing 180–220 g were used in the pharmacokinetic study. The rats were fasted overnight before dosing. Every treatment group contain 3 rats. Rats were dosed with SKLB-TB1001 or BTZ043 suspension at 5 mg/kg (i.v. and p.o.). Compounds were dissolved in 5% DMSO/10% Solutol HS15 in saline for intravenous injection and suspended in 0.5% CMC for oral administration. Blood was collected from the jugular vein of each animal at the following times after administration of drugs: 0.083, 0.25, 0.5, 1, 2, 4, 6, 8 and 24 h after a single i.v. dosing; and 0.25, 0.5, 1, 2, 4, 6, 8 and 24 h after a single oral dosing. All blood samples were centrifuged at 3000 r/min for 10 min to obtain serum which was then stored at −20 °C. 150 μL of the serum was added to 500 μL of acetonitrile and the mixture was centrifuged at 13000 r/min for 10 min to remove protein. The supernatant was dried and dissolve in 100 μL of acetonitrile, the solution was centrifuged at 13000 r/min for 10 min. The supernatant was moved to a sample bottle for HPLC analysis. Total area under the concentration time curve (AUC), the elimination half-time (t_1/2_), the peak concentration (C_max_) and the time to reach peak concentration (T_max_) of samples were determined directly from the experimental data using WinNonlin V6.2.1.

### *In vivo* acute toxicity study

All animal experiments have been approved by the Institutional Animal Care and Treatment Committee of Sichuan University in China and were carried out in accordance with the approved guidelines. 20 BALB/c mice in both sexes were administered with SKLB-TB1001 CMC suspension at 1 g/kg by intraperitoneal injection, control mice remained untreated. Animals were continuously observed for 24 hours after administration. In each group, numbers of deaths and general behavior were recorded within 2 weeks, LD_50_ values were calculated. 2 weeks later, animals were sacrificed, blood samples were collected from the eye vein for biochemical and hematological tests. The organs and tissues of animals were removed and fixed in 10% formalin and embedded in paraffin for histological evaluation.

### Statistical analysis

Statistical analysis was analyzed by 2-tailed Student’s t test. In all statistical analysis, P values < 0.05 were considered to be statistically significant.

## Additional Information

**How to cite this article**: Gao, C. *et al*. Benzothiazinethione is a potent preclinical candidate for the treatment of drug-resistant tuberculosis. *Sci. Rep*. **6**, 29717; doi: 10.1038/srep29717 (2016).

## Supplementary Material

Supplementary Information

## Figures and Tables

**Table 1:** Antimycobacterial activity of benzothiazinethione SKLB-TB1001 *in vitro*.

Compound	MIC (μg/mL)			IC_50_ (μg/mL)			SI^a^
	H37Rv	*M. avium*	Erdman	Vero	A549	J774	IC_50_/MIC
SKLB-TB1001	0.02	32	<0.05	>100	>100	>32	>2167
BTZ043	0.02	32	<0.05	>100	25	32	539
PBTZ169	0.02	/	≤0.05	>100	/	/	/
INH	0.47	16	N.D.	>100	N.D.	N.D.	

INH, isoniazid; SI, selectivity index.

^a^IC_50_ of A549/MIC against *Mtb* H37Rv.

**Table 2:** Activity of benzothiazinethione against drug-resistant TB clinical isolates.

Strains		SM	INH	RMP	EMB	OFLX	LVFX	MOX	AMK	KM	TB1001
H37Rv		1	0.03	<0.03	1	0.5	0.25	0.06	0.5	1	<0.0156
MDR-TB	M3	64	8	64	4			N.D.			<0.0156
	M4	8	1	64	2						<0.0156
	M8	64	4	64	4						<0.0156
	M18	64	1	64	8						<0.0156
XDR-TB	X15	8	16	64	16	8	4	2	64	64	<0.0156
	X17	64	1	64	16	32	32	8	64	64	<0.0156

SM, streptomycin; EMB, ethambutol; KM, kanamycin; INH, isoniazid; RMP, rifampicin; LVFX, levo-ofloxacin; OFLX, ofloxacin; MOX, moxifloxacin; CPM, capreomycin; AMK, amikacin. M3, 4, 8 and 15 are clinical isolated multidrug-resistant strains. X18 and 75 are clinical isolated extensively drug-resistant strains. N.D. indicates not determined.

**Table 3:** Interactions of SKLB-TB1001 and existing TB drugs against *M*. *tuberculosis* H37Rv *in vitro*.

	MIC against H37Rv (μg/ml)				FICI	Interaction
	MIC alone		MIC in combo.			
1001 + RMP	TB1001	RMP	TB1001	RMP	0.22	Synergistic
	0.02	0.006	0.001	0.001		
1001 + INH	TB1001	INH	TB1001	INH	0.68	Additive
	0.02	0.03	0.007	0.01		
1001 + MOX	TB1001	MOX	TB1001	MOX	0.97	Additive
	0.02	0.037	0.008	0.021		
1001 + LZD	TB1001	LZD	TB1001	LZD	1.34	Additive
	0.02	0.145	0.013	0.1		
BTZ043 + RMP	BTZ043	RMP	BTZ043	RMP	0.84	Additive
	0.011	0.06	0.008	0.007		

LZD, Linezolid; FICI, Fractional inhibitory concentrations index.

MABA checkerboard assays were used to characterize interaction of benzothiazinethione with existing TB drugs.

**Table 4:** Oral pharmacokinetic parameters of compounds in SD rats.

Comp.	C_max_ (ng/ml)	T_max_ (h)	T_1/2_ (h)	AUC (ng * h/mL)	F (%)
TB1001	193	1.13	1.45	847	44.4
BTZ043	543	0.25	1.22	899	29.5

C_max_, maximum plasma concentration; T_max_, time of peak plasma concentration; T_1/2_, terminal half-life for elimination; AUC, area under the curve (t = 0 to infinity); F, absolute oral bioavailability. F = AUC (p.o., 5 mg/kg)/AUC(i.v., 5 mg/kg).

**Table 5:** Bacterial burden in mouse lungs.

	Log_10_ CFU (SD)				
	Untreated	Vehicle	RMP	TB1001	BTZ043
Day-3	2.90 (2.40)	N.D.	N.D.	N.D.	N.D.
Day-10	3.72 (3.25)	N.D.	N.D.	N.D.	N.D.
Day-36	N.D.	6.27 (5.85)	3.73 (3.19)	2.84 (2.93)	6.31 (5.85)

Log_10_ colony forming unit (CFU) counts in lungs was determined by calculating the bacteria loads in organs from the untreated group and treated groups. Mean log_10_ CFU per lung (SD) are presented. N.D. indicates not determined.

**Table 6:** Blood chemistry analysis of acute toxicity test (Mean ± SE, n = 5).

Parameters	Male Control	Male Treatment	Female Control	Female Treatment
ALB (g/L)	31.84 ± 0.55	32.84 ± 0.44	33.08 ± 0.50	32.18 ± 0.65
ALP (U/L)	169.40 ± 3.82	159.20 ± 6.98	168.00 ± 1.14	162.00 ± 4.70
AST (Ug/L)	139.40 ± 6.59	153.40 ± 4.30	154.60 ± 3.71	154.60 ± 5.57
BUN (mmol/L)	7.70 ± 0.38	7.10 ± 0.60	7.52 ± 0.22	7.06 ± 0.35
CHOL (mmol/L)	3.21 ± 0.10	3.31 ± 0.07	3.31 ± 0.07	3.31 ± 0.08
TP (g/L)	70.24 ± 2.76	68.38 ± 1.58	69.58 ± 0.90	68.56 ± 1.42

ALB: albumin; ALP: alkaline; phosphatase; AST: aspartate aminotransferase; BUN: blood urea nitrogen; CHOL: cholesterol esters; TP: total proteins.
