# Supplementary material for: Benzothiazinethione is a potent preclinical candidate for the treatment of drug-resistant tuberculosis
Source: Sci Rep. 2016 Jul 13;6:29717. doi: 10.1038/srep29717 (PMC4942819; doi:10.1038/srep29717)
Supplement: Supplementary Information [file srep29717-s1.doc]

**Supplementary information**

**benzothiazinethione is a potent preclinical candidate for the treatment of drug-resistant tuberculosis**

Chao Gao^1^,**^+^**, Cuiting Peng^1,2^,**^+^**, Yaojie Shi^1^, Xinyu You^1,2^, Kai Ran^1^, Lu Xiong^1^, Ting-hong Ye^1^, Lidan Zhang^1^, Ningyu Wang ^1^,Yongxia Zhu^1^, Kun Liu^3^, Weiqiong Zuo^1^, Luoting Yu^*,1^ & Yuquan Wei^1^.

^1^State Key Laboratory of Biotherapy/ Collaborative Innovation Center for Biotherapy, West China Hospital, West China Medical School, Sichuan University, Chengdu, 610041, Sichuan, China.

^2^Department of Pharmaceutical and Bioengineering, School of Chemical Engineering, Sichuan University, Chengdu, 610041, Sichuan, China.

^3^Department of Respiratory and Critical Care Medicine, West China Hospital, Sichuan University, Chengdu, 610041, Sichuan, China.

**^+^**These authors contributed equally to this work.

**Synthetic route and procedure for the synthesis of benzothiazinethione**

**Supplementary Table S1-S3**

**Supplementary Figures S1-S3**

**Synthetic route and procedure for the synthesis of benzothiazinethione**

Reagents and conditions: (a) (COCl)_2_, CH_2_Cl_2_, DMF (cat), room temp (rt), 2 h; (b) NH_4_NCS, CH_2_Cl_2_, PEG-400 (cat), rt, 2-4 h; (c) 1,4-dioxa-8-azaspiro[4.5]decane, rt., 90 min; (d) Lawesson’s reagent, toluene, reflux, 1 h.

3g of 2-chloro-3-nitro-5-(trifluoromethyl) benzoic acid were suspended in 20ml dry dichloromethane, 2.64ml of Oxalyl Chloride and 0.05ml DMF were added successively to the stirred suspension. The reaction was stirred at room temperature for 2h, and evaporated in vacuo. The residue was dissolved in 25ml dry dichloromethane and added to 1.57g of ammonium thiocyanate dropwise under stirring. 0.2g of PEG-400 was added to the suspension, it was then stirred at room temperature for 1.5h. The reaction mixture was filtered and the filtrate was added to 1.49g of 1,4-dioxa-8-azaspiro[4.5]decane over 20min. The reaction mixture was stirred at room temperature until completion of the reaction according to TLC. The reaction was quenched by adding 30ml water, the mixture was stirred for 30min and filtered. The filter residue (benzothiazineone) was dried under reduced pressure and then suspended in 100ml dry toluene, 4.22g of Lawesson’s reagent were added, the reaction mixture was refluxed until consumption of the starting material according to TLC. The mixture was cooled to room temperature, filtered, the filtrate was concentrated under reduced pressure. The residue was purified over silica gel using EA/PE(1:8,v/v) as eluent to obtain the red product(2.11g, 46.9%).Purity of compounds was>98% as determined by HPLC. mp 203.3-205.5°C.

^1^H NMR (DMSO-*d_6_*, 400MHz): *δ* 9.27(s, 1H), 8.81(s, 1H), 4.05(d, *J*=7.2Hz, 4H), 3.90(s, 4H), 1.83(s, 4H);

^13^C NMR (Chloroform-*d_6_*, 400MHz): *δ* 166.53, 161.80, 143.92, 134.14, 133.35, 129.66(q, *J*=35.35), 126.63, 126.03, 122.38(q, *J*=273.71), 106.21, 64.76, 44.68, 35.12 .

ESI-MS: m/z 456.0 (M+Na)^+^.

**Supplementary Table S1: Structure, MIC and cytotoxicity of the** **benzothiazinethiones**

|  | **Substitution** | | **MIC (μM)** | **IC_50_ (μM)** |
| --- | --- | --- | --- | --- |
| **Comp.** | **R1** | **R2** |  |  |
| **5a** | **NO2** |  | **12.5** | **>20** |
| **5b** | **NO2** |  | **25** | **>20** |
| **5c** | **NO2** |  | **6.3** | **>20** |
| **5d** | **CF3** |  | **12.5** | **>20** |
| **5e** | **CF3** |  | **<0.1** | **>20** |
| **5f** | **CF3** |  | **<0.1** | **>20** |
| **5g**  **(SKLB-TB1001)** | **CF3** |  | **<0.1** | **>20** |
| **BTZ043** |  |  | **<0.1** | **>20** |

Compound MIC was conducted by ShangHai Chempartner Co., Ltd (Shanghai, China) using the microplate Alamar Blue assay (MABA) against *Mycobacterium tuberculosis* H37Ra (ATCC 25177). Cytotoxicity of tested compounds was assessed on Vero cells.

**Supplementary Table S2: ADME/T profiling data for** **benzothiazinethiones and benzothiazineone *in vitro***

|  | SKLB-TB1001 | 5f | BTZ043 |
| --- | --- | --- | --- |
| Solubility^a^( PH=6.5) | <1 | <1 | <1 |
| Lipophilicity (ClogP) | 1.74 | 2.45 | 4.91 |
| Plasma protein binding (M) (%) | 96.2 | >99 | >99 |
| Permeability (Mean Papp, MDR1) |  |  |  |
| A to B (10^-6^ cm/s) | 6.96 | 0.11 | 9.02 |
| B to A (10^-6^ cm/s) | 7.27 | 0.10 | 7.17 |
| Efflux Ratio | 1.04 | 0.89 | 0.79 |
| CYP inhibition (IC50, µM) |  |  |  |
| CYP3A4 | >10 | >10 | >10 |
| CYP2C19 | >10 | >10 | >10 |
| CYP2C9 | >10 | >10 | >10 |
| CYP2D6 | >10 | >10 | >10 |

The *In vitro* ADME/T assays were conducted at WuXi AppTec Co. Ltd.(Shanghai). ClogP was calculated with ChemDraw Ultra 14.0. In the study of bi-directional permeability across MDR1-MDCKII cell monolayer, permeability was correlated with the apparent permeability coefficient Papp (cm/s), which was interpreted as follows: Low permeability( Papp ≤ 1.0×10^-6^ cm/s), Moderate permeability(1.0×10^-6^ < Papp < 5.5×10^-6^ cm/s) or High permeability( Papp ≥ 5.5×10^-6^ cm/s). ^a^aqueous solubility at room temperature.

**Supplementary Table S3: Intracellular activity of SKLB-TB1001 against *Mtb* Erdman**

|  | Log_10_ CFU | S.D. | Fold(log) reduction |
| --- | --- | --- | --- |
| D0 | 5.15 | 4.75 | -- |
| D7 | 6.85 | 6.23 | -- |
| RMP | 6.44 | 5.98 | 0.41 |
| SKLB-TB1001 | 4.77 | 4.03 | 2.08 |
| BTZ043 | 4.70 | 4.40 | 2.15 |

Fold reduction represents the decrease of log_10_ CFU counts between D7 and treatment groups.

CFU, colony forming unit.

**Supplementary Table S4: Checkerboard synergy assay of SKLB-TB1001 with RMP**

|  |  |  | RMP conc. (μg/ml) | | | | | |  |
| --- | --- | --- | --- | --- | --- | --- | --- | --- | --- |
|  |  |  | 0.05 | 0.017 | 0.006 | 0.002 | 0.0007 | 0.0002 |  |
|  |  | TB1001 only |  |  |  |  |  |  | RMP only |
| TB1001 conc. (μg/ml) | 0.05 | 100.49% | 100.00% | 99.96% | 100.09% | 100.23% | 98.72% | 99.16% | 100.93% |
|  | 0.017 | 89.11% | 100.00% | 100.04% | 100.04% | 98.38% | 99.61% | 99.46% | 100.34% |
|  | 0.006 | 83.26% | 100.04% | 100.00% | 99.75% | 98.23% | 100.00% | 98.57% | 90.05% |
|  | 0.002 | 45.88% | 98.87% | 100.05% | 100.03% | 97.34% | 99.16% | 95.47% | 87.13% |
|  | 0.0007 | 8.09% | 99.16% | 100.04% | 99.95% | 91.16% | 88.31% | 66.84% | 84.21% |
|  | 0.0002 | 7.65% | 99.75% | 100.00% | 99.99% | 86.31% | 82.23% | 72.89% | 59.46% |

**Supplementary Figure S1**


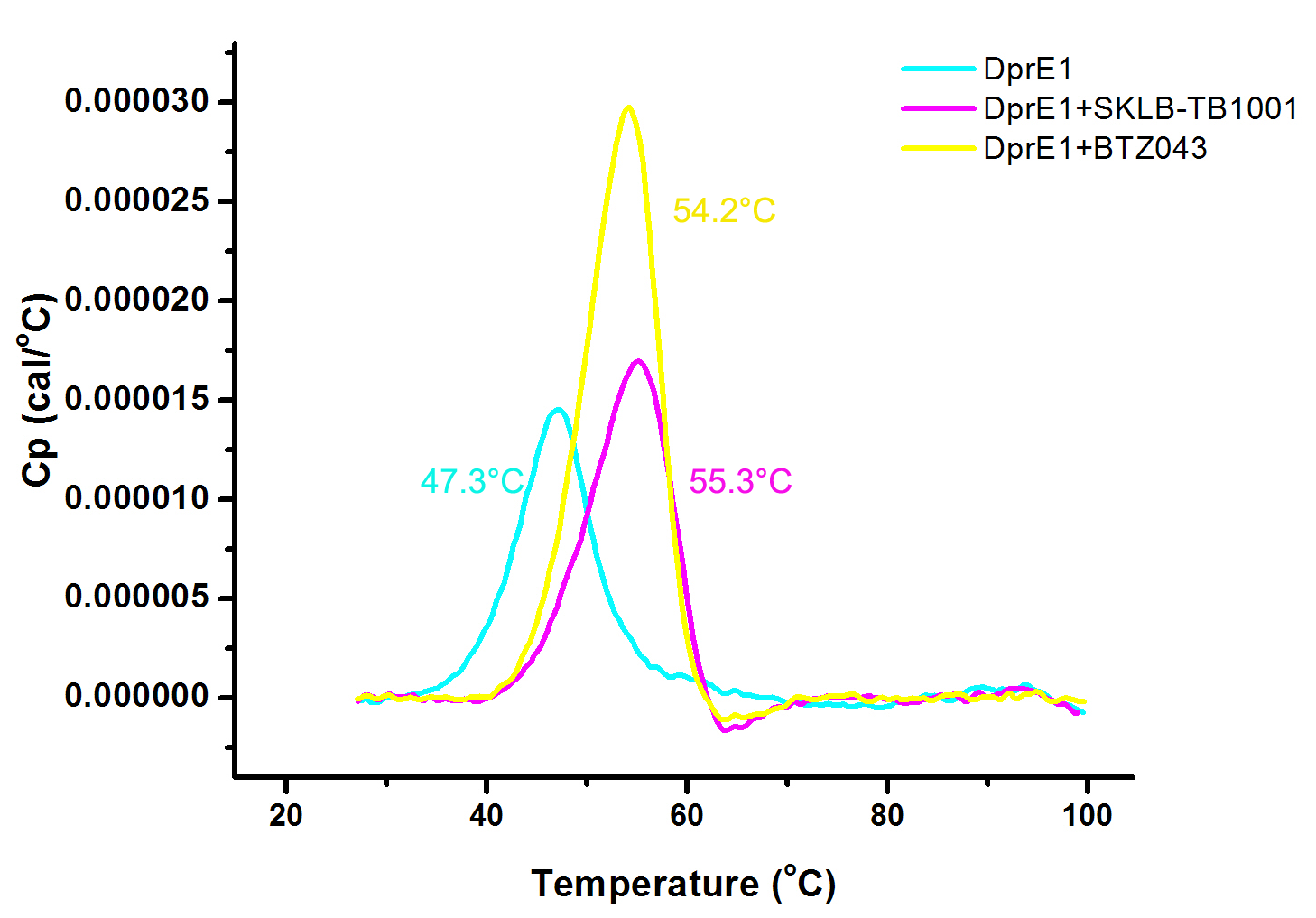


**Supplementary Figure 1. The experimental DSC curve of DprE1 with SKLB-TB1001.** DSC was successfully used for probing the structural [stabiliti](../../../../C:/Documents%20and%20Settings/Administrator/Local%20Settings/Application%20Data/Yodao/DeskDict/frame/20160217135335/javascript:void(0)%3B)es through denaturation temperature (*T_m_*) increases due to inhibitors’ strong binding to DprE1 in the presence of the substrate FPR. The parameter *ΔT_m_* is defined by the difference between denaturation temperature of the pure protein and the protein incubate with each inhibitor. The *T_m_* of the pure DprE1 was 47.3 °C, while it decreased to 55.3 °C and 54.2 °C when in the presence of SKLB-TB1001 and BTZ043. The *ΔT_m_* was 8.0 °C and 6.9 °C respectively.

**Supplementary Figure S2**


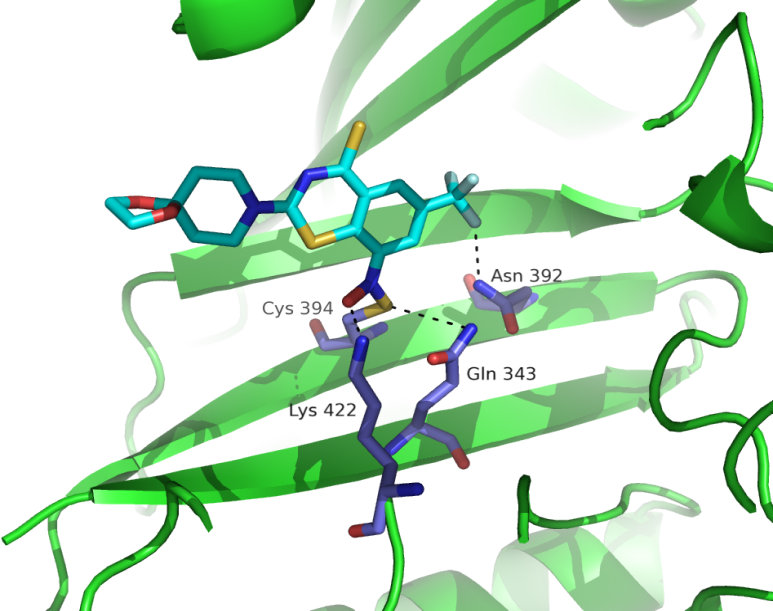

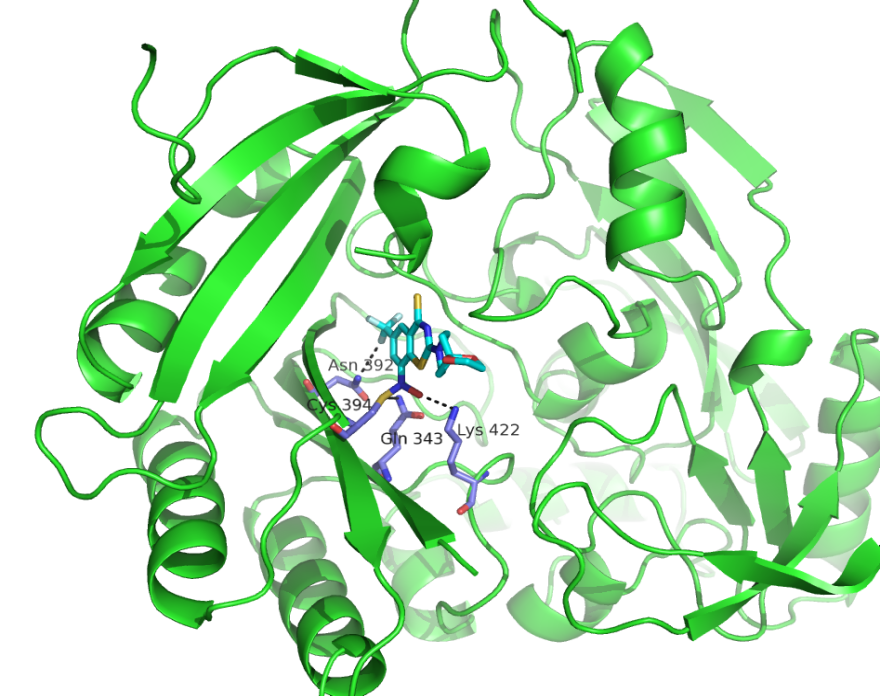


**Supplementary Figure 2. The computational docking analysis of SKLB-TB1001 with DprE1.**The *M. smegmatis* DprE1 crystal structure (PDB #4F4Q) was used for the docking study. The nitroso intermediate of SKLB-TB1001 (carbons are cyan) formed a semimercaptal adduct with Cys394 of DprE1 though [covalent](../../../../C:/Documents%20and%20Settings/Administrator/Local%20Settings/Application%20Data/Yodao/DeskDict/frame/20160217135335/javascript:void(0)%3B) [binding](../../../../C:/Documents%20and%20Settings/Administrator/Local%20Settings/Application%20Data/Yodao/DeskDict/frame/20160217135335/javascript:void(0)%3B). There were also other interactions between SKLB-TB1001 with other residues (Lys422, Gln324, Asn392) seen from the docking model (right). The docking pattern for SKLB-TB1001 with DprE1 is similar to BTZ043 with DprE1, both showed strong interaction with the enzyme.

**SupplementaryFigureS3**


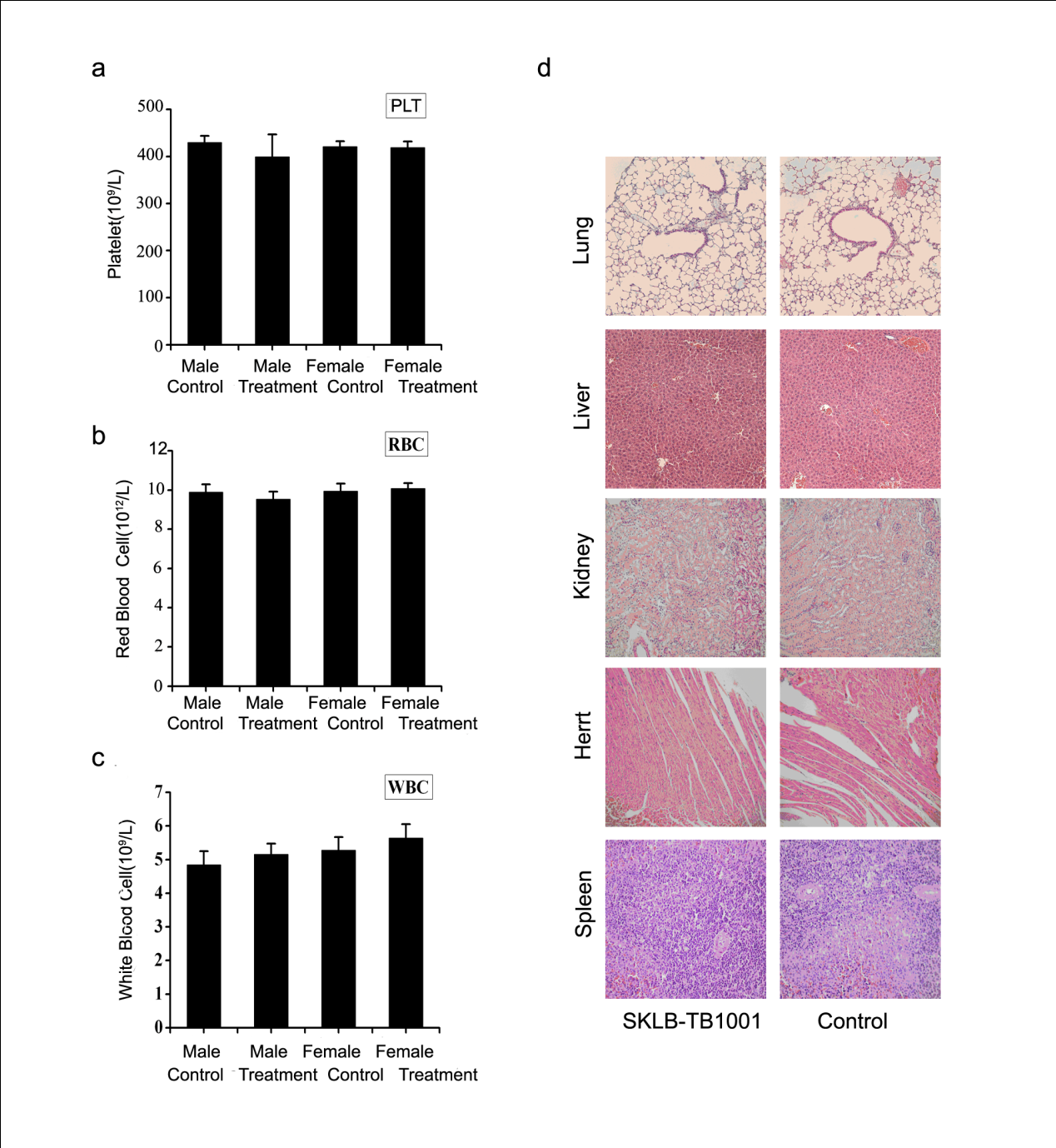


**Supplementary Figure 3. Hematological parameters and histological changes of treated mice in the acute toxicity test.** (a)~(c) Complete blood count of acute toxicity test .(a) Platelet.(b) Red blood cell.(c) White blood cell.(d) Examples of H&E-stained histological sections of mice heart, liver, spleen, lung, and kidney of SKLB-TB1001 group (left row) and control group (right row), respectively.
